# Design and implementation of a novel pharmacogenetic assay for the identification of the CYP2D6*10 genetic variant

**DOI:** 10.1186/s13104-022-05993-6

**Published:** 2022-03-16

**Authors:** Nadeeka Dimuthu Kumari Ranadeva, Nirmala Dushyanthi Sirisena, Tithila Kalum Wetthasinghe, Nafeesa Noordeen, Vajira Harshadeva Weerabaddana Dissanayake

**Affiliations:** Department of Anatomy, Genetics and Biomedical Informatics, Faculty of Medicine, University of Colombo, Colombo 08, Sri Lanka

**Keywords:** Breast cancer, Tamoxifen, CYP2D6*10, Pharmacogenetics, Genotypes

## Abstract

**Objectives:**

Tamoxifen is considered to be the most widely used adjuvant therapy for hormone receptor positive breast cancer in premenopausal women. However, it is reported that nearly 30% of patients receiving tamoxifen therapy have shown reduced or no benefits. This may be due to the high inter-individual variations in the *CYP2D6* gene that is involved in tamoxifen metabolism. The CYP2D6*10 gene variant (rs1065852C>T) is reported to be commonly found in Asian and South Asian populations. The present study was undertaken to design a novel pharmacogenetic assay (Single step-Tetra Arms Polymerase Chain Reaction) for the identification of the CYP2D6*10 variant and implement the designed assay by genotyping a cohort of breast cancer patients.

**Results:**

The novel assay was successfully designed, optimized and validated using Sanger sequencing. Blood samples from 70 patients were genotyped. The following bands were observed in the gel image: Control band at 454 bp; band for C allele at 195 bp; band for T allele at 300 bp. The genotype frequencies for the CYP2D6*10 (rs1065852C>T) variant were: CC-24.28% (17/70), CT-75.71% (53/70), TT-0% (0/70). The allele frequencies were: T-allele-37.86% and C-allele-62.14%.

**Supplementary Information:**

The online version contains supplementary material available at 10.1186/s13104-022-05993-6.

## Introduction

Breast cancer is the most common cancer among women and overall, it is the second most common cancer in the world [1]. In Sri Lanka, it is the most common cancer among females with a prevalence rate of 27% [2]. Although tamoxifen has been the drug of choice to treat hormone receptor positive breast cancer in premenopausal women, nearly a one third of patients treated with adjuvant tamoxifen therapy do not obtain the desired benefits. It is reported that there is a substantial inter-individual variability in the response to this drug and the beneficial and adverse effects also appear to be variable and unpredictable for individual patients [3]. Variants in the *CYP2D6* gene have been reported to affect the metabolism of tamoxifen in breast cancer patients resulting in variable responses to the drug due to reduced enzyme activity. Tamoxifen is converted to its more active form endoxifen by the CYP2D6 enzyme [4]. However, due to marked variability in the *CYP2D6* genotypes among population groups, it has been difficult to formulate a common guideline for using the genotype as a determinant factor in prescribing tamoxifen. Although many pharmacogenomic studies have been conducted to evaluate the possibility of incorporating *CYP2D6* genotyping into the therapeutic management of hormone receptor positive breast cancer, conflicting results have posed as a barrier to achieving this. In order to support the establishment of pharmacogenomic testing for tamoxifen therapy in breast cancer patients, it is fundamental to establish prevalence data of the most common variants in under-represented populations. Existing data reports CYP2D6*10:rs1065852C > T as one of the most common variants in the South Asian and Sri Lankan populations that is known to affect tamoxifen metabolism. Knowledge of the prevalence of this variant would be of importance to gain the maximum benefit associated with the use of this drug [5]. The current study was undertaken to design a novel pharmacogenetic assay for easy detection of the CYP2D6*10:rs1065852C>T variant and implement the designed assay by genotyping a cohort of Sri Lankan breast cancer patients.

## Main text

### Methods

The study was conducted at the Human Genetics Unit, Faculty of Medicine, University of Colombo. It was an experimental study, where a novel assay was designed for the targeted variant, and a cohort of hormone receptor positive breast cancer patients were genotyped for the CYP2D6*10 variant using the optimized and validated assay. An existing resource of stored venous blood samples obtained from 70 hormone receptor positive breast cancer patients which were collected into EDTA tubes and stored at  − 20 °C were used for the present study.

The primers for the CYP2D6*10 rs1065852 variant were newly designed using web-based tools. *CYP2D6* gene sequence was obtained from the NCBI (*CYP2D6* ID: 1565). The Primer1 is a special software designed for obtaining primer tetrads that are specific for T-ARMS PCR technique [6]. The Primer1 software was operated through the web interface where an input of a target DNA sequence is enabled. The maximum size of the DNA sequence is up to 1000 bases in the 5′ to 3′ direction. Serial cloner enabled cutting a 1000 bp fragment (which included the variant in the middle) that was inserted to Primer1. Other parameters were also considered for primer design: the desired minimum, maximum and average melting temperatures (°C) for a primer oligo; the millimolar concentration of salt and the nanomolar concentration of annealing oligos in the PCR (this is used in the calculation of T_m_—melting temperature); the % GC content (the minimum and maximum allowable for any primer); the primer lengths and the minimum and maximum complementarity and product sizes [7]. In par with the selected parameters, the program generated primer sequence sets. To select the optimum primer sets the melting temperatures were considered for each primer set (inner and outer). NCBI primer blast was used to check the specificity of the primers. The details of the designed set of primers, and their product sizes are given in Table 1.

**Table 1:** Details of the designed primers for the variant CYP2D6*10

Primers	Sequence (5′ to 3′)	Length (bp)	Product Size (bp)	T_m_ (°C) primer blast result	T_m_ (°C)
CYP2D6*10 (rs1065852 C>T)					
CYP2D6*10-CF	5′TGGCAGCACAGTCAACACAGCAGGTTC 3′	27	Control: 454 (T): 300 (C): 195	69.49	64.9
CYP2D6*10-CR	5′CTGGTCCAGCCTGTGGTTTCACCCAC 3′	26		69.26	65.2
CYP2D6*10-F(C)	5ʹAACGCTGGGCTGCACGCTCCC 3′	21		68.11	67.7
CYP2D6*10-R(T)	5′GGCAGTGGCAGGGGGCCTGGGGA 3′	23		73.83	72.6

Genomic DNA was extracted using QIAamp^®^ DNA mini kits (Qiagen Ltd., UK) according to the manufacturer’s protocol. The Thermal Cycler (Bio-Rad) was used to conduct gradient PCR for the targeted variant CYP2D6*10. The primer annealing temperatures mentioned in the primer vials by the manufacturer were considered for the annealing temperature gradient in the first attempt at PCR optimization. After a series of optimization attempts, the final optimized cyclic conditions for this variant consisted of an initial denaturation of 3 min at 94 °C (initial denaturation), followed by 35 PCR cycles of 1 min at 94 °C (denaturation), 30 s at 65 °C (annealing temperature), 1 min at 72 °C and the final extension of 5 min at 72 °C. The optimized PCR master mixture contained 5.0 µL of 5 × Green Go-Taq Flexi Buffer, 1.0 µL of MgCl_2_ (25 mM), 1.0 µL dNTP mixture (2.5 mM each), 1.5 µL of 12.5 mM CYP2D6*10-CF, 1.5 µL of 12.5 mM CYP2D6*10-CR, 1.0 µL of 12.5 mM CYP2D6*10-F (C) and 0.75 µL of 12.5 mM CYP2D6*10-R (T), 1.5 µL of 5 mM Betaine and dH_2_O. 8 μL of the PCR products of each protocol were separated based on 2% agarose gel at 80 V for 1 h and visualized by ethidium bromide under a UV illuminator.

Four randomly selected samples were validated by Sanger sequencing. The Sanger validated samples were used as controls to compare the genotyping results of the remaining samples [under Additional file information, Additional file 1: Figure S1A (homozygous wild type), Figure S1B (heterozygous variant), Figure S1C (homozygous wild type) and Figure S1D (homozygous wild type) show the Sanger sequencing electropherogram images of the variant].

The bands for each genotype were observed in the gel images: Control band at 454 bp; band for C allele at 195 bp; band for T allele at 300 bp. The genotype frequencies were determined using the data (allele counts) observed from the gel images. They were systematically recorded, and genotype frequencies for homozygous wild-type (CC), heterozygous variant (CT), and homozygous variant (TT) genotypes and the allele frequencies for C (wild type) and T (variant) alleles were calculated using an online calculator (available from: https://wpcalc.com/en/equilibrium-hardy-weinberg/) and tested for Hardy‑Weinberg equilibrium. Standard descriptive statistics were used to analyze the demographic data.

### Results

All patients in the sample cohort that was genotyped were females with hormone receptor positive breast cancer. Their mean age was 60.12 ± 6.56 years.

T-ARMS PCR protocol was successfully designed and implemented for the identification of the CYP2D6*10 variant. The expected gel band patterns were observed in the gel image results (Control band: 454 bp; band for C allele: 195 bp; band for T allele: 300 bp). Under Additional file information, Additional file 2: Figure S2 shows the expected gel band patterns for the targeted variant.

**Fig. 1:**
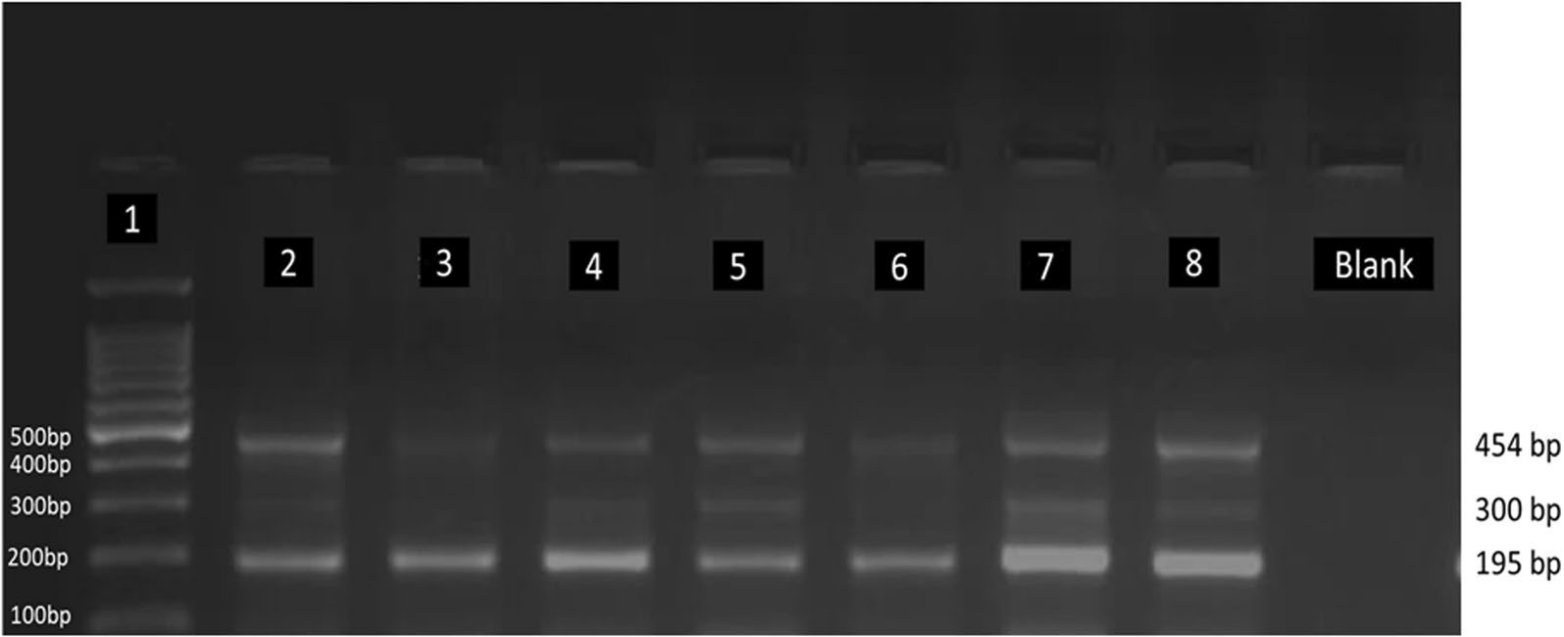
Gel band patterns of CYP2D6*10. Gel picture of T-ARMS PCR of CYP2D6*10 100 C>T genotyping showing control band at 454 bp (Lane 2, 3, 4, 5, 6, 7, 8), variant allele at 300 bp (Lane 2, 4, 5, 7, 8), wild type allele at 195 bp (Lane 2, 3, 4, 5, 6, 7, 8 and 100 bp ladder (Lane 1). Samples in Lane 2 (C/T) and Lane 3 (C/C) were confirmed with Sanger sequencing

Figure 1 shows an actual gel image of the genotype results.

The gel images of all 70 patient samples (with the sample numbers indicated in table) are given in Additional file 3: Figure S3.

The genotype and allele frequencies are shown in Table 2.

**Table 2:** Genotype and allele frequencies of CYP2D6*10 variant

Samples (n = 70)	Genotype Frequency *n* (%)			Major allele frequency (%)	Minor allele frequency (%)
Hormone receptor positive breast cancer patients	CC	CT	TT	C	T
	17 (24.3%)	53 (75.7%)	0	0.6214 (62.1%)	0.3786 (37.9%)

Out of the 70 samples which were genotyped, homozygous wild type (CC) and heterozygous (CT) samples were detected but no homozygous samples were detected for the variant T allele (Table 2). The predominant genotype was CT (75.7%) and the minor allele frequency of the CYP2D6*10 variant in this cohort was 37.9%. The results of all the genotyped samples are shown in Additional file 4: Table S1.

### Discussion

The T-ARMS PCR is a technique which many researchers are beginning to adopt as it is a cost-effective method compared to other techniques that are used to detect SNVs [8, 9]. However, the optimization step can be time-consuming and laborious [9]. The presence of GC rich regions near the SNV of interest might be a limiting factor for the use of this technique [10]. One of the common problems faced with T-ARMS PCR is the specificity, where the MgCl_2_ concentration, primer ratio, and dNTP ratio can play a vital part in the optimization process [10]. Different gradients of MgCl_2_, dNTP and annealing temperatures were used during the optimization process in this study. The use of Betaine [10] and Dimethyl sulfoxide (DMSO) [11] is also commonly reported across studies that have used this technique. In this study, Betaine was used in a lower concentration and volume during the optimization process.

The CYP2D6*10 variant has been studied in different populations and the present study is an addition to this data from the South Asian region. The genotyping results obtained were comparable to data available from a previous Sri Lankan study conducted by Tharanga et al*.* in 2013, where the CYP2D6*10 minor allele frequency in a Sri Lankan population (n = 75) was reported to be 39%, similar to the 37.9% obtained in the cohort of 70 hormone receptor positive breast cancer patients genotyped in this study. The study by Tharanga et al*.* further identified that the minor allele frequency was highest among the Moors and 50% of the participants in that study were males [12]. The current study did not enumerate the ethnic specific variations since the ethnicity of the breast cancer patients was not available for subgroup analysis.

### Conclusions

A novel T-ARMS PCR assay was successfully designed, optimized and validated to genotype the CYP2D6*10 variant and could be implemented as a cost-effective technique. Homozygous wild type (CC) and heterozygous variant (CT) alleles in CYP2D6*10 were found in the genotyped samples. Homozygotes for the variant allele were not identified in this cohort.

### Limitations

- The T-ARMS PCR assay development is challenging specially when the target region has a high GC percentage which could lead to prolongation of the optimization process.
- With the use of Betaine, the intensity of the control and other bands may reduce, this could cause difficulty in detecting the relevant bands on the gel image.
- Primer dimer formation.
- The small sample size used for genotyping in this study. A larger study would be ideal for frequency estimation of the targeted variant in the population.

## Supplementary Information

**Additional file 1: Figures S1A**, **S1B**, **S1C** and **S1D**: Sanger confirmation of CYP2D6*10 homozygous wild type (CC) and heterozygous variant (CT) samples. **S1A** Homozygous wild type sample. **S1B** Heterozygous variant type sample. **S1C** Homozygous wild type sample. **S1D** Homozygous wild type sample

**Additional file 2: Figure S2.** Gel band patterns of CYP2D6*10 variant

**Additional file 3: Figure S3.** Gel images of all genotyped samples for CYP2D6*10 variant

**Additional file 4: Table S1.** Genotyping results and age of the hormone receptor positive breast cancer patient cohort

## Data Availability

All the datasets generated and or analyzed during the current study are available in the Additional file section (Additional file 4: Table S1, Additional file 1: Figure S1, Additional file 3: S3). The following databases were accessed to obtain the details and FASTA sequence related to the gene of interest: NCBI—Gene—*CYP2D6*—https://www.ncbi.nlm.nih.gov/gene/1565; NCBI RefSeq—*CYP2D6*—https://www.ncbi.nlm.nih.gov/nuccore/NG_008376.4?report=fasta; SNPedia—*CYP2D6* gene—https://www.snpedia.com/index.php/CYP2D6.

## Abbreviations

*CYP2D6*: Cytochrome P450 2D6
T-ARMS PCR: Tetra Primer Amplification Refractory Mutation System based Polymerase Chain Reaction
PCR: Polymerase Chain Reaction
DNA: Deoxyribonucleic acid
SNV: Single Nucleotide Variant
TE: Tris EDTA
NCBI: National Center for Biotechnology Information
BLAST: Basic Local Alignment Search Tool
CF: Control Forward
CR: Control Reverse
DMSO: Dimethyl sulfoxide
F (C): Forward primer for C allele
R (T): Reverse primer for T allele
A: Adenine
G: Guanine
C: Cytosine
T: Thymine
