# Supplementary figures and images for: Design and implementation of a novel pharmacogenetic assay for the identification of the CYP2D6*10 genetic variant
Source: BMC Res Notes. 2022 Mar 16;15:104. doi: 10.1186/s13104-022-05993-6 (PMC8925205; doi:10.1186/s13104-022-05993-6)

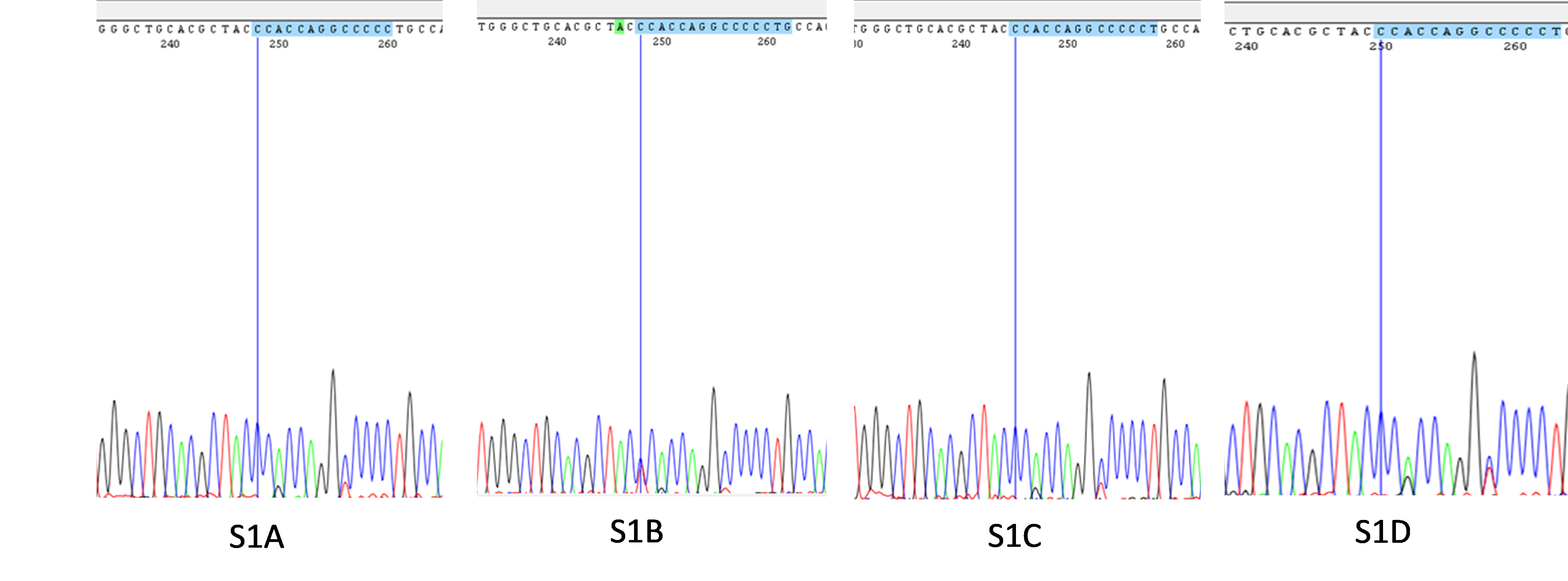

Supplement: Supplementary file 1 — Additional file 1: Figures S1A, S1B, S1C and S1D: Sanger confirmation of CYP2D6*10 homozygous wild type (CC) and heterozygous variant (CT) samples. S1A Homozygous wild type sample. S1B Heterozygous variant type sample. S1C Homozygous wild type sample. S1D Homozygous wild type sample [file 13104_2022_5993_MOESM1_ESM.png]

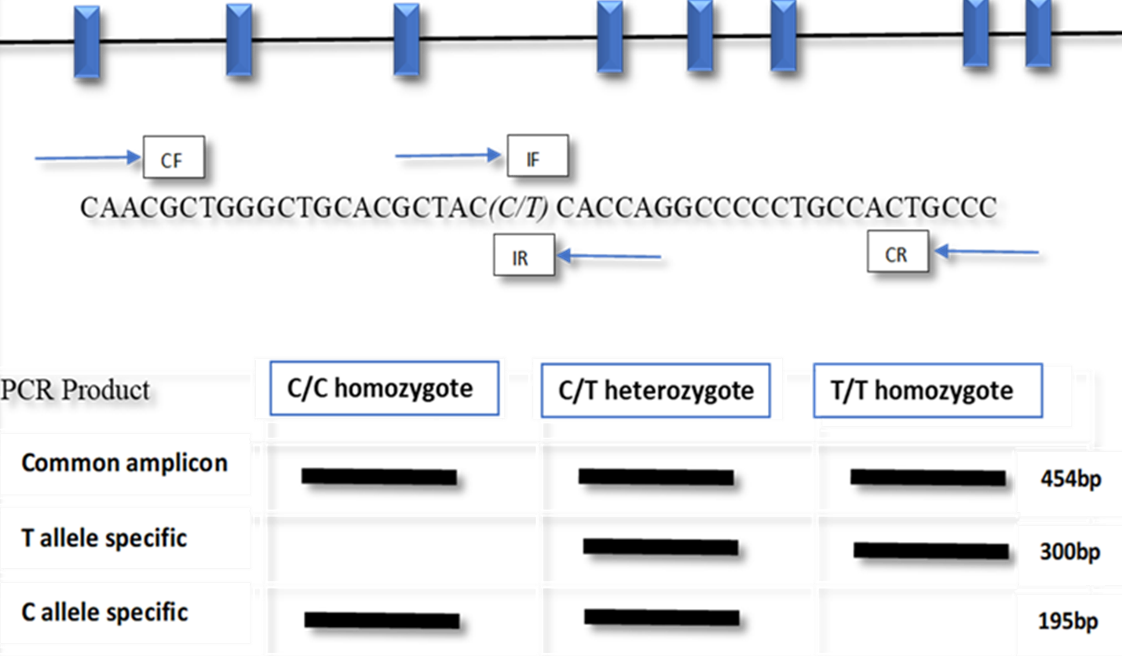

Supplement: Supplementary file 2 — Additional file 2: Figure S2. Gel band patterns of CYP2D6*10 variant [file 13104_2022_5993_MOESM2_ESM.png]

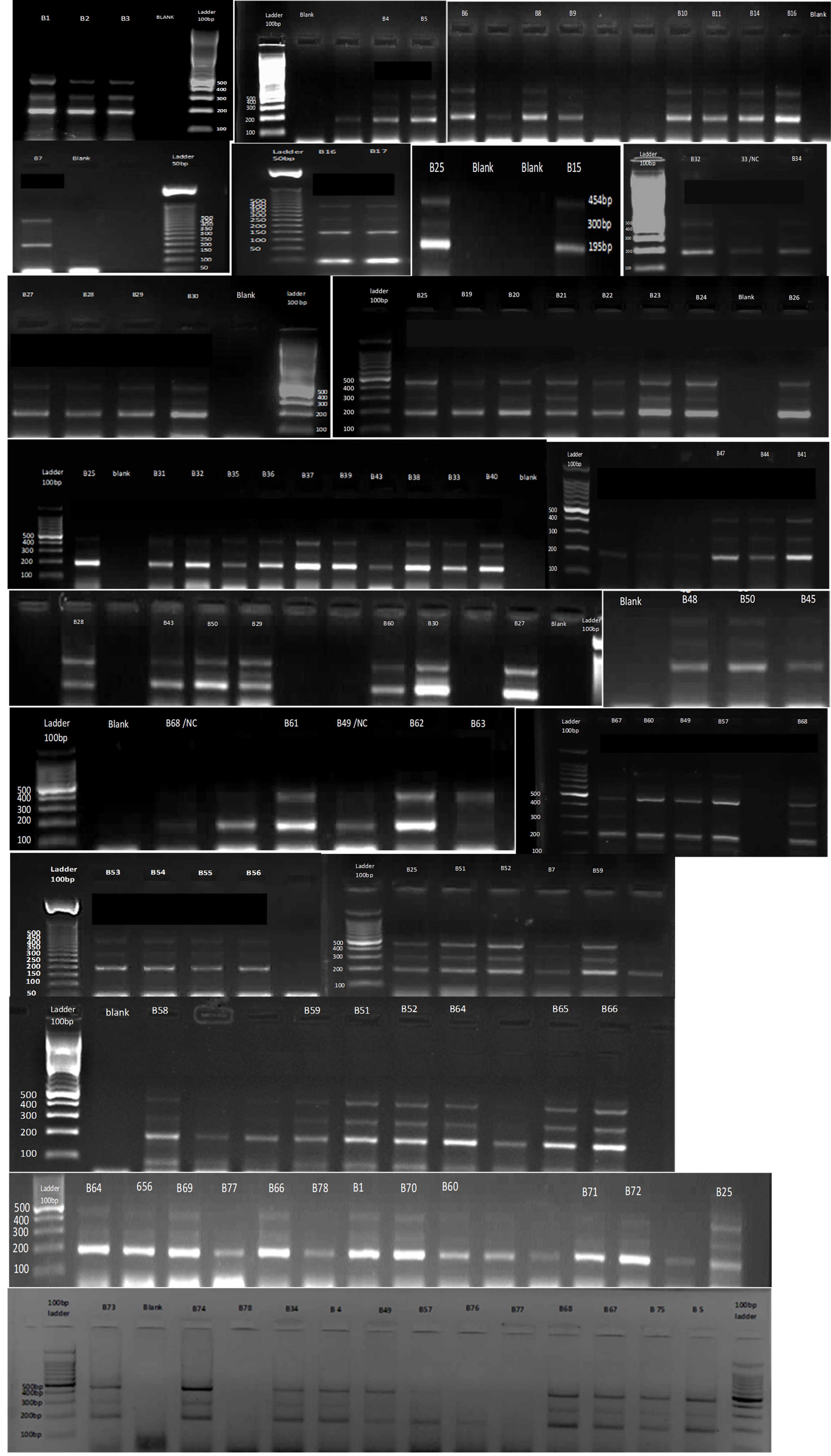

Supplement: Supplementary file 3 — Additional file 3: Figure S3. Gel images of all genotyped samples for CYP2D6*10 variant [file 13104_2022_5993_MOESM3_ESM.png]
